# Response to letter, ‘Immortal time bias in retrospective analysis'

**DOI:** 10.1038/bcj.2015.55

**Published:** 2015-07-31

**Authors:** M A Dimopoulos, A S Swern, J S Li, M Hussein, L Weiss, Y Nagarwala, R Baz

**Affiliations:** 1 Department of Clinical Therapeutics, University of Athens School of Medicine, Athens, Greece; 2 Department of Biostatistics, Celgene Corporation, Summit, NJ, USA; 3 Department of Medical Affairs, Celgene Corporation, Summit, NJ, USA; 4 Department of Drug Safety, Celgene Corporation, Summit, NJ, USA; 5 Department of Hematologic Malignancies, H Lee Moffitt Cancer Center and Research Institute, Tampa, FL, USA

We appreciate the response from Hsieh *et al.*^1^ to our publication, ‘Efficacy and safety of long-term treatment with lenalidomide and dexamethasone in patients with relapsed/refractory multiple myeloma'.^2^ As a part of our original study, we assessed the effect of lenalidomide and dexamethasone treatment on ‘humoral improvement' in multiple myeloma patients, which was defined as an increase in uninvolved immunoglobulin A levels from baseline. We found that, compared with humoral nonresponders whose mean immunoglobulin A levels remained unchanged, humoral responders to lenalidomide and dexamethasone treatment showed improvements in long-term outcomes such as progression-free survival (PFS) and overall survival (OS), as determined by log-rank tests (*P*<0.0001 for PFS and OS; Figures 2a and b). In the letter by Hsieh *et al.*, ‘Immortal time bias in retrospective analysis',^1^ the group questions the use of log-rank tests (or Cox regression models using response as a time-independent covariate), and remarks that this could have resulted in ‘immortal time bias'.^3^ In this context, this means that a bias towards improved outcomes may have occurred in the humoral responder patient group, dependent on the longer observation time in that group. In other words, patients who remained alive and progression-free had a higher chance of achieving a humoral response.

Humoral response rate was 79% in patients experiencing long-term benefit of therapy (PFS ⩾3 years) versus other patients (54% *P*=0.006); however, median time to humoral response was 1 month in both groups. To address the concerns of Hsieh *et al.*,^1^ we reanalyzed PFS and OS using humoral response as a time-dependent covariate, as suggested. Following reanalysis, humoral response in patients receiving lenalidomide and dexamethasone was again found to be a highly significant determinant of patient outcome. Comparing humoral responders and nonresponders revealed significant differences in PFS (hazard ratio 0.59, 95% confidence interval 0.43–0.82; *P*=0.0016) and OS (hazard ratio 0.55, 95% confidence interval 0.40–0.76; *P*=0.0003). Humoral responders had a 41% reduction in the risk of progression or death and a 45% risk reduction in death.

This time-dependent analysis confirms that humoral response significantly predicts the long-term benefit of lenalidomide and dexamethasone therapy for patients with relapsed/refractory multiple myeloma, in agreement with the original conclusions of the paper.

## Notes

MAD has had a consultant role for, and has received honoraria from, Celgene. ASS, JSL, MH, LW and YN are Celgene employees and have equity ownership. RB has received research funding from Celgene.

## References

[bib1] Hsieh P-Y Liu C-J Teng C-J Immortal time bias in retrospective analysis: comment on ‘Efficacy and safety of long-term treatment with lenalidomide and dexamethasone in patients with relapsed/refractory multiple myeloma' Blood Cancer J 2015 5 e283 25723854 10.1038/bcj.2015.4 PMC4349258

[bib2] Dimopoulos MA Swern AS Li JS Hussein M Weiss L Nagarwala Y Efficacy and safety of long-term treatment with lenalidomide and dexamethasone in patients with relapsed/refractory multiple myeloma Blood Cancer J 2014 4 e257 25382609 10.1038/bcj.2014.77 PMC4571985

[bib3] Shariff SZ Cuerden MS Jain AK Garg AX The secret of immortal time bias in epidemiologic studies J Am Soc Nephrol 2008 19 841 843 18322159 10.1681/ASN.2007121354

